# 血液病患者同基因造血干细胞移植后植入综合征的发生率及临床特征

**DOI:** 10.3760/cma.j.issn.0253-2727.2023.04.005

**Published:** 2023-04

**Authors:** 瑞 娄, 兰平 许, 晓辉 张, 开彦 刘, 昱 王, 晨华 闫, 晓军 黄, 于谦 孙

**Affiliations:** 北京大学人民医院血液科，北京大学血液病研究所，国家血液系统疾病临床医学研究中心，北京 100044 Peking University People's Hospital, Peking University Institute of Hematology, National Clinical Research Center for Hematologic Disease, Beijing 100044, China

**Keywords:** 同基因造血干细胞移植, 植入综合征, 临床表现, Syngeneic hematopoietic stem cell transplantation, Engraftment syndrome, syngeneic, Clinical picture

## Abstract

**目的:**

探究血液病患者同基因造血干细胞移植（syn-HSCT）后植入综合征（ES）的发生率及其临床特征。

**方法:**

纳入1994年1月至2018年5月在北京大学人民医院接受syn-HSCT的21例患者，对其临床资料进行回顾性分析。

**结果:**

①21例患者中男13例，女8例，中位年龄24（4～46）岁。重型再生障碍性贫血2例，骨髓增生异常综合征1例，急性髓系白血病8例，急性淋巴细胞白血病6例，慢性髓性白血病4例。移植后中位随访时间为817（24～5 602）d。②21例syn-HSCT患者中7例（33.3％）发生ES。首次出现ES相关症状的中位时间为移植后8（5～13）d，诊断ES的中位时间为移植后10（7～14）d。所有ES患者均以糖皮质激素治疗并获得完全缓解，中位症状持续时间为2（1～5）d。③多因素分析显示，原发病为急性髓系白血病（*HR*＝15.298，95％*CI* 1.486～157.501，*P*＝0.022）和中性粒细胞植入时间<12 d（*HR*＝17.459，95％*CI* 1.776～171.687，*P*＝0.014）是ES发生的独立危险因素。ES组与未发生ES组移植后总生存率与无病生存率差异无统计学意义。

**结论:**

血液病患者syn-HSCT后具有较高的ES发生率，但预后较好。

## Abstract (en)

**Objective:**

To explore the incidence and clinical characteristics of engraftment syndrome（ES）after syngeneic hematopoietic stem cell transplantation（syn-HSCT）in patients with hematological diseases.

**Methods:**

The clinical data of 21 patients who received syn-HSCT at People's Hospital of Peking University from January 1994 to May 2018 were retrospectively analyzed.

**Results:**

Seven（33.3％）of 21 patients developed ES. The onset of ES symptoms occurred at a median of 8（range: 5–13）days after HSCT, and the diagnosis of ES occurred at a median of 10（range: 7–14）days after HSCT. Steroids were administered immediately after the diagnosis of ES, the median time of symptom continuance was 2（range: 1–5）days, and all patients showed complete resolution of ES symptoms. In the multivariate analysis, patients with acute myeloid leukemia and faster neutrophil reconstitution were the risk factors for ES（*HR*＝15.298, 95％ *CI* 1.486–157.501, *P*＝0.022, and *HR*＝17.459, 95％ *CI* 1.776–171.687, *P*＝0.014）. Meanwhile, there was no significant difference in the overall survival and disease-free survival between patients with ES and those without ES.

**Conclusion:**

A high incidence of ES was observed in syn-HSCT recipients. Moreover, the prognosis of ES was excellent.

植入综合征（Engraftment syndrome, ES）是造血干细胞移植（HSCT）后早期并发症，常发生在中性粒细胞植入前后，以非感染性发热、类似急性移植物抗宿主病（aGVHD）的皮疹和非心源性肺水肿为主要临床表现，可伴有低氧血症、肺部浸润、体液潴留、体重增加、器官功能障碍或中枢神经系统病变等临床表现[1]–[7]。

1995年Lee等[8]首次报道自体造血干细胞移植（auto-HSCT）后发生ES，此后在各种类型HSCT中均有报道，在auto-HSCT中的发生率为6.9％～25.8％[6],[9]–[10]，在allo-HSCT中的发生率为13.0％～22.1％[7],[11]–[13]，在全相合allo-HSCT中的发生率为2.0％～10.1％[1],[14]，在单倍体HSCT（haplo-HSCT）中的发生率约为21.9％[14]，在脐血干细胞移植中的发生率为26.8％～77.0％[5],[15]–[17]，在同基因造血干细胞移植（syn-HSCT）中的发生率高达46.9％[18]。因syn-HSCT病例数量较少，对syn-HSCT后ES的研究不多，国内没有相关报道。本研究对北京大学血液病研究所syn-HSCT病例进行回顾性分析，探讨ES的发生率及其临床特征。

## 病例与方法

一、病例资料

本研究纳入1994年1月至2018年5月在北京大学人民医院接受syn-HSCT的21例血液病患者（均为首次HSCT），对其临床资料进行回顾性分析。随访截止日期为2020年7月12日。供者基因型经限制性片段长度多态性聚合酶链反应（PCR-RFLP）或短串联重复序列（STR）技术确认。

二、诊断标准及定义

依据文献[9],[18]结果，我们对以下病例进行了分析：①所有在粒细胞植入前7 d至植入后7 d内出现非感染性发热（新出现的体温≥38 °C、微生物培养阴性且无抗生素反应记录）的患者；②没有发热，但有其他明显ES临床特征（类似GVHD的皮疹、非感染性腹泻、低氧血症、非心源性肺水肿等）的患者。影像学也用于评估有无感染迹象。腹泻：新出现或从基线开始增加的每天≥500 ml或≥2次的液状大便且没有感染证据。皮疹：类似急性GVHD的皮疹且无药物反应或潜在感染的证据。缺氧：氧饱和度<95％。肝功能障碍：转氨酶>2倍正常上限或总胆红素>20 mg/L。肺部浸润经胸部X线或CT扫描证实，在没有感染、心力衰竭或肺栓塞的临床和实验室证据时，可判定为ES相关。

符合Spitzer[2]或Maiolino[3]诊断标准，或虽不符合上述标准但经临床医师认为ES是最可能的原因者可临床诊断为ES。

三、预处理及移植资料

采用我中心常规预处理方案[19]。18例患者以白消安（Bu）联合环磷酰胺（Cy）为基础的改良Bu/Cy预处理方案，2例患者采用以Cy联合兔抗人胸腺细胞免疫球蛋白（ATG）为基础的Cy/ATG预处理方案，1例患者给予全身放射治疗（TBI）联合Cy为基础的TBI/Cy预处理方案。移植物均为粒细胞集落刺激因子（G-CSF）动员的骨髓（或）外周血干细胞，采集的供者干细胞直接经中心静脉回输。

中性粒细胞绝对计数（ANC）≥0.5×10^9^/L连续3 d为粒细胞植入；PLT≥20×10^9^/L连续7 d且脱离血小板输注为血小板植入。

总生存（OS）期定义为从干细胞回输后第1天至随访截止或因任何原因出现死亡的时间；无病生存（DFS）期定义为从干细胞回输后第1天至随访截止、疾病血液学复发或任何原因死亡的时间。

四、统计学处理

应用SPSS 22.0软件进行数据分析。对各种临床参数进行描述性分析；计量资料按照中位数进行分组；计数资料的比较采用Fisher精确检验；采用Cox回归分析寻找ES发生的相关因素（单因素和多因素分析）。将单因素分析中有统计学意义的因素纳入多因素分析。生存分析采用Kaplan-Meier曲线进行分析。*P*<0.05为差异有统计学意义。

## 结果

一、患者一般临床资料

21例患者中男13例，女8例，中位年龄24（4～46）岁。中位随访时间为移植后817（24～5602）d。重型再生障碍性贫血（SAA）2例，骨髓增生异常综合征（MDS）1例，急性髓系白血病（AML）8例，急性淋巴细胞白血病（ALL）6例，慢性髓性白血病（CML）4例。回输单个核细胞（MNC）中位数为6.25（1.68～10.25）×10^8^/kg，CD34^+^细胞中位数为2.48（0.74～5.78）×10^6^/kg（只收集到15例患者的数据）。21例患者中7例（33.3％）发生ES，14例（66.7％）未发生ES，发生、未发生ES两组患者的临床特征详见表1。

**表1:** 同基因造血干细胞移植后发生、未发生植入综合征（ES）血液病患者的临床特征比较（例）

指标	未发生ES组（14例）	ES组（7例）	*P*值
移植年龄			0.063
<24岁	9	1	
≥24岁	5	6	
性别			1.000
男	9	4	
女	5	3	
诊断			0.146
AML	3	5	
ALL	5	1	
MDS	1	0	
AA	1	1	
CML	4	0	
移植前疾病状态			1.000
低风险（CR1、CP、AA、MDS）	12	6	
高风险（≥CR2）	2	1	
干细胞来源			
骨髓	7	1	
外周血干细胞	2	0	
骨髓+外周血干细胞	5	6	
移植预处理方案			1.000
改良Bu/Cy	12	6	
TBI/Cy	1	0	
Cy/ATG	1	1	
GVHD预防			0.255
有	4	0	
无	10	7	
GVHD预防方案			1.000
CsA	1	0	
MTX	1	0	
CsA+MTX	1	0	
CsA+MTX+MMF	1	0	
无	10	7	
单个核细胞回输量			0.361
<6.25 ×10^8^/L	8	2	
≥6.25 ×10^8^/L	6	5	
CD34^+^细胞回输^a^			1.000
<2.48 ×10^6^/L	4	3	
≥2.48 ×10^6^/L	4	4	
应用G-CSF			0.624
是	4	1	
否	10	6	
粒细胞植入时间			0.017
<12 d	2	5	
≥12 d	12	2	
血小板植入时间			0.156
<9 d	3	4	
≥9 d	11	3	

**注** ALL：急性淋巴细胞白血病；AML：急性髓系白血病；MDS：骨髓增生异常综合征；AA：再生障碍性贫血；CML：慢性髓性白血病；CR1、CR2：第1、2次完全缓解；CP：慢性期；Bu：白消安；Cy：环磷酰胺；TBI：全身放射治疗；ATG：兔抗人胸腺细胞免疫球蛋白；CsA：环孢素A；MTX：甲氨蝶呤；MMF：霉酚酸酯。^a^6例早期患者无CD34^+^细胞数据

二、造血重建及ES发生情况

移植后中位中性粒细胞植入时间为移植后12（8～28）d，中位血小板植入时间为移植后9（7～62）d。21例患者中有7例（33.3％）发生ES，其中1例（14.3％）符合Spitzer诊断标准，3例（42.9％）符合Maiolino诊断标准，另有3例（42.9％）不符合上述两项诊断标准，但是经过临床医师的仔细判读，认为ES是其最临床症状最可能的解释。首次出现ES相关症状的中位时间为移植后8（5～13）d，中位ES诊断时间为移植后10（7～14）d。

ES的临床表现：非感染性发热5例（71.4％）、皮疹4例（57.1％）、腹泻5例（71.4％）、低氧血症1例（14.3％）、转氨酶升高1例（14.3％）。其中表现为发热伴腹泻、低氧血症、转氨酶升高1例（14.3％），发热腹泻伴皮疹1例（14.3％），发热伴腹泻2例（28.6％），发热伴皮疹1例（14.3％），腹泻伴皮疹1例（14.3％），单纯皮疹1例（14.3％）。详见表2。

**表2:** 7例同基因造血干细胞移植后发生植入综合征（ES）血液病患者的临床资料

例号	性别	年龄（岁）	原发病	ES临床表现	ES治疗	治疗结果
1	男	10	SAA	发热、皮疹	地塞米松5 mg/d	完全缓解
2	男	37	ALL	腹泻、皮疹	地塞米松7.5 mg/d	完全缓解
3	女	31	AML	发热、腹泻	地塞米松2.5 mg/d	完全缓解
4	女	46	AML	发热、腹泻	甲泼尼龙1 mg·kg^−1^·d^−1^	完全缓解
5	男	33	AML	皮疹	甲泼尼龙1 mg·kg^−1^·d^−1^	完全缓解
6	男	24	AML	发热、皮疹、腹泻	甲泼尼龙1 mg·kg^−1^·d^−1^	完全缓解
7	女	44	AML	发热、腹泻、非心源性肺水肿、肝功能异常、低氧血症	甲泼尼龙200 mg/d	完全缓解

**注** SAA：重型再生障碍性贫血；ALL：急性淋巴细胞白血病；AML：急性髓系白血病

三、发生ES的危险因素

多因素分析显示，原发病为AML（*HR*＝15.298，95％*CI* 1.486～157.501，*P*＝0.022）和中性粒细胞植入时间<12 d（*HR*＝17.459，95％*CI* 1.776～171.687，*P*＝0.014）是ES发生的独立危险因素。详见表3。

**表3:** 影响ES发生的Cox单因素和多因素分析结果

因素	单因素分析			多因素分析		
	*HR*	95%*CI*	*P*值	*HR*	95%*CI*	*P*值
性别（女，男）	1.378	0.308~6.169	0.675			
移植年龄（<24岁，≥24岁）	0.127	0.015~1.070	0.058			
原发病（AML，其他）	6.710	1.273~35.373	0.025	15.298	1.486~157.501	0.022
移植前疾病状态（低风险，高风险）	0.919	0.110~7.650	0.938			
造血干细胞来源（骨髓，外周血±骨髓）	0.221	0.027~1.841	0.163			
MNC回输量（<6.25×10^8^/L，≥6.25×10^8^/L）	0.337	0.065~1.749	0.196			
CD34^+^细胞回输量（<2.48×10^6^/L，≥2.48×10^6^/L）	0.857	0.191~3.843	0.840			
ATG应用（有，无）	1.325	0.159~11.034	0.794			
GVHD预防（有，无）	0.033	0.000~67.836	0.382			
CsA应用（有，无）	0.037	0.000~201.581	0.453			
中性粒细胞植入时间（<12 d，≥12 d）	8.052	1.537~42.187	0.014	17.459	1.776~171.687	0.014
血小板植入时间（<9 d，≥9 d）	2.846	0.635~12.759	0.172			
G-CSF应用（有，无）	0.556	0.067~4.629	0.587			

**注** AML：急性髓系白血病；MNC：单个核细胞；ATG：免抗人抗胸腺细胞免疫球蛋白；GVHD：移植物抗宿主病；CsA：环孢素A；G-CSF：粒细胞集落刺激因子。低风险包括急性白血病第一次完全缓解（CR1）、慢性髓性白血病慢性期（CP）、再生障碍性贫血（AA）、骨髓增生异常综合征（MDS），高风险是指第二次及以上完全缓解（≥CR2）

四、ES的治疗

7例ES患者均在发生上述症状时应用广谱抗生素治疗，并在诊断ES后即给予肾上腺皮质激素治疗。其中5例患者使用甲泼尼龙（1 mg·kg^−1^·d^−1^或200 mg/d）治疗，2例患者使用地塞米松（2.5 mg/d或7.5 mg/d）治疗。诊断ES应用肾上腺皮质激素治疗后症状持续的中位时间为2（1～5）d。所有ES患者经治疗后症状均完全缓解。

五、复发与生存

随访截至2020年7月12日，中位随访时间为移植后817（24～5602）d。21例患者中9例（40.9％）复发，3例患者死亡（均死于复发）。ES组、未发生ES组移植后2年OS率分别为（66.7±19.2）％、（87.5±11.7）％（*P*＝0.366）（图1A），DFS率分别为（53.6±20.1）％、（68.8±15.3）％（*P*＝0.362）（图1B）。

**图1:**
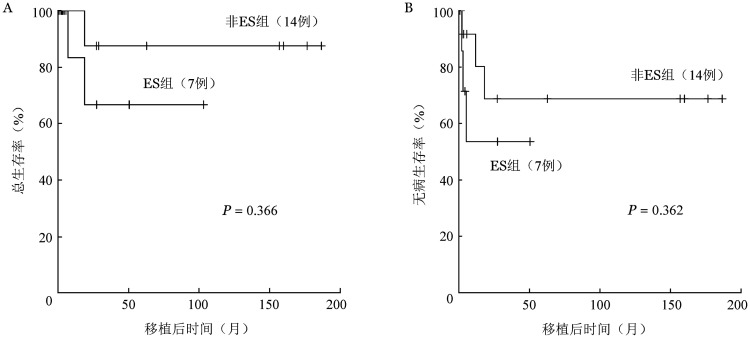
同基因造血干细胞移植后发生、未发生植入综合征（ES）血液病患者移植后总生存（A）、无病生存（B）曲线

## 讨论

本组病例ES的发生率为33.3％，低于Koreth等[18]的报道（46.9％）。由于两项研究病例数均较少，无法进行直接比较。但造成差异的原因可能包括：首先，Koreth等的研究中包含淋巴瘤及多发性骨髓瘤患者，同时中位年龄高于本组病例，移植前疾病状态多数为高风险（87.5％），且大部分患者采用以TBI为基础的预处理方案（56.3％），并且所有患者均未给予GVHD预防。

目前ES发生机制尚未完全阐明。研究显示移植前预处理可造成内皮细胞损伤并促进炎症因子释放，且ES的发生与粒细胞植入同步，多种炎症因子（如IL-5、IL-6、TNF-α和IFN-γ）在ES早期明显升高[11],[20]，提示ES可能由活化的白细胞和多种细胞因子及其他炎症因子介质释放引起的促炎状态引起。然而，目前在ES患者中并没有发现具有预测意义的细胞因子异常。ES发生的危险因素可能包括：auto-HSCT主要包括女性、移植前化疗史[9]；脐血干细胞移植主要包括疾病危险分层低风险、清髓预处理、应用CsA、未应用MTX预防GVHD、未应用糖皮质激素预防GVHD、MNC回输量>5.43×10^8^/L [15],[17]；haplo-HSCT包括疾病危险分层高风险 [14]；全相合allo-HSCT主要危险因素是血液系统恶性肿瘤[1]。本研究结果显示，AML患者和中性粒细胞快速植入（<12 d）是ES发生的独立危险因素。这可能与ES发生的病理生理机制有关，ES的发生涉及多种细胞和细胞因子的相互作用，中性粒细胞可能在某种程度上有助于ES的发生[10],[21]–[22]。同时，既往研究显示移植前治疗可能与ES的发生有关[22]–[23]，这可能是本研究中AML患者是ES发生的危险因素的部分原因。本研究病例数少，上述结论尚需进一步研究探讨。

以往研究显示，ES的临床症状包括：非感染性发热（91％～100％）、非药物性皮疹（38％～100％）、腹泻（28％～95.5％）、肺水肿（13.3％～72.7％）、体重增加（0～81.8％）、肝肾功能异常（12％～53.3％）和脑病（3％～10％）[4]–[6],[15]–[16],[18]。

粒细胞植入前与植入后均可发生ES，在不同研究中其发生时间略有不同。早期文献报道ES常发生在粒细胞植入前后96 h内[2]或外周血中首次出现中性粒细胞前后24 h内[3]。近期研究发现其可发生于粒细胞植入1周前[16]–[17]。本研究中各临床症状的发生率的较文献报道有所下降，发生时间大体一致，一方面与病例数较少有关，另一方面是随着对ES认识的深入，早期干预可能缩短了疾病的进程。

糖皮质激素（甲泼尼龙1～2 mg/kg）作为ES的首选治疗方案已在临床上广泛应用，但目前尚未明确治疗反应与剂量之间的关系[4]。77％～100％的患者对糖皮质激素有反应且在1～4 d内症状缓解[1],[7]，治疗后出现新的症状或复发的时间为2（1～7）d[7]。本研究7例ES患者在糖皮质激素治疗后症状均得到完全缓解，其中5例患者使用甲泼尼龙（1 mg/kg或200 mg/d）治疗，2例患者使用地塞米松（2.5 mg/d或7.5 mg/d）治疗，糖皮质激素治疗后症状持续的中位时间为2（1～5）d，提示目前糖皮质激素的起始剂量与其应用疗程可能存在一定相关性。
